# Prevalence of autoantibodies in patients with juvenile idiopathic arthritis: results from the German inception cohort ICON-JIA

**DOI:** 10.1186/s12969-022-00668-9

**Published:** 2022-02-02

**Authors:** Caroline Schulz, Sabrina Fuehner, Bernhard Schlüter, Manfred Fobker, Claudia Sengler, Jens Klotsche, Martina Niewerth, Kirsten Minden, Dirk Foell

**Affiliations:** 1 Department of Paediatric Rheumatology and Immunology, University Hospital Muenster, Albert-Schweitzer-Campus 1, Building D3, 48149 Muenster, Germany; 2 Centre of Laboratory Medicine, University Hospital Muenster, Muenster, Germany; 3 Epidemiology Unit, German Rheumatism Research Center, Berlin, Germany; 4 Department of Pediatric Respiratory Medicine, Immunology and Critical Care Medicine, Charité Medical University Berlin, Rüdesheimer Str. 50, 14197 Berlin, Germany

## Abstract

**Background:**

An association of different autoimmune diseases is suspected. In juvenile idiopathic arthritis (JIA), only few and partially conflicting data on the co-existence of other autoimmune disorders are available. The prevalence of autoantibodies in patients with JIA in Germany is not known.

**Methods:**

Samples from 499 patients (median age at time of blood collection 11 years, median disease duration 4.4 years) in the prospective, multicenter inception cohort of children newly diagnosed with JIA (ICON-JIA) were analysed for the presence of anti-thyroid antibodies, celiac disease-specific antibodies (anti-tTG IgA, anti-tTG IgG), and connective tissue disease-associated antibodies (CTD-screen).

**Results:**

A total of 76 (15.2%) patients had either clinically diagnosed autoimmune comorbidity or elevated autoantibodies. Of 21 patients with clinical autoimmune comorbidity, only 8 were also serologically positive at the time of testing, while 55 patients had autoantibodies without clinical diagnosis. Thus, 63 patients (12.6%) had at least one elevated autoantibody. Antibodies against thyroglobulin were found in 3% and against thyreoperoxidase in 4% of the samples. TSH receptor antibodies could not be detected in any of the 499 patients. Tissue transglutaminase antibodies were elevated in 0.4% of the patients. A positive screen for CTD-specific antinuclear antibodies was found in 7%, but only rarely specific antibodies (anti-dsDNA 1.4%, anti-SS-A and -SS-B 0.2% each, anti-CENP-B 0.4%) were confirmed.

**Conclusions:**

In our study, a specific correlation between JIA and other autoimmune phenomena could not be confirmed. The lack of well-matched control groups makes interpretation challenging. Further data need to corroborate the suspected increased risk of developing other autoimmune phenomena in JIA patients.

**Supplementary Information:**

The online version contains supplementary material available at 10.1186/s12969-022-00668-9.

## Background

Juvenile idiopathic arthritis (JIA) is the most common chronic rheumatic disease in childhood. According to the current International League of Associations for Rheumatology (ILAR) classification, 7 categories can be differentiated based on clinical and laboratory parameters [1]. The pathogenesis is unclear, but it is often referred to as autoimmune arthritis, especially for oligoarthritis and seropositive and negative polyarthritis.

The co-occurrence of JIA with other autoimmune disease is a matter of debate [2]. However, individual studies come to different results regarding the prevalence of autoimmune diseases in JIA patients, so that screening examinations are not routinely carried out. This can partly be explained by the fact that autoimmune diseases are initially asymptomatic. They develop over a long period of time, while laboratory markers that can indicate the presence of an autoimmune disease are often only used for diagnosis when irreversible tissue damage has already occurred [3].

Data from a single-center analysis in Italy with 79 patients showed that 15.2% of JIA patients had at least one autoimmune disease in addition to JIA. Autoimmune thyroid disease was found to be most common (10.1%) [4]. Another study (*n* = 151) reported a 7-fold increased risk for celiac disease and a high prevalence of autoimmune thyroiditis (11.9%) together with a high rate of subclinical hypothyroidism (9.3%) in JIA [5]. In an Austrian study, JIA patients (*n* = 95) were found to have a 14-fold increased risk of developing celiac disease [6]. A large cross-sectional study using two United States administrative healthcare claims databases compared the prevalence of multiple autoimmune diseases of more than 29,000 JIA patients with that of more than 134,000 matched children with attention deficit hyperactivity disorder (ADHD). Almost all investigated autoimmune diseases were more prevalent in patients with JIA, and especially psoriasis and uveitis were significant comorbidities [7]. Similar findings were reported from a comparison of patients with JIA with a control group from the general pediatric patient population at the Cincinnati Children’s Hospital Medical Center [8]. Also a German study showed, that type 1 diabetes is significantly more frequent in patients with JIA [9].

On the other hand, there are also studies showing that other autoimmune diseases, especially celiac disease, are not more prevalent in JIA patients than in the normal population. In a Dutch study, 62 children with JIA were tested for celiac disease. With a prevalence of 1.5%, the results were close to the prevalence of the normal population (Dutch children) [10]. A study from Iran also tested 53 children for anti-tTG IgA (anti-tissue transglutaminase), of which only one child (1.8%) had elevated levels [11]. Another study found no child with elevated anti-tTG levels among 96 JIA patients [12].

The aim of our cross-sectional study was to quantify the presence of autoantibodies in patients with established JIA. We used serum samples from the biobank of the prospective, multicenter inception cohort of children newly diagnosed with JIA (ICON-JIA) in Germany to analyse thyroid and celiac disease-specific antibodies, as well as antibodies with reasonable specificity for connective tissue disorders. Age and gender differences as well as other influencing variables were taken into account.

## Methods

### Study design

ICON-JIA (Inception Cohort of Newly diagnosed patients with juvenile idiopathic arthritis) is a prospective, longitudinal and controlled observational study of early JIA in 11 participating pediatric rheumatology centers in Germany [13]. It was funded from 2009 through 2022 by the Federal Ministry of Education and Research (BMBF) and approved by the ethics committees of the Charité Medical University and the University of Muenster. More than 950 children and adolescents with JIA and almost 490 controls were finally recruited from 2010 to 2013 after providing informed consent. The patients were regularly examined by pediatric rheumatologists and serum samples were taken when laboratory investigations were indicated. For ethical reasons, venipuncture was not performed solely for autoantibody profiles. Paediatric rheumatologists provided information on disease activity, concomitant diseases (asked by means of a tick-box list of predefined conditions every 3 months in the first year of observation and then every 6 months) and treatment. Furthermore, the study participants and their parents were regularly interviewed and completed questionnaires regarding disease activity, functional capacity, compliance, quality of life and satisfaction, as well as other disease-related factors. All samples were shipped to the laboratory at the University of Muenster, aliquoted and stored at − 80 °C. The samples were primarily used for measurements of disease-related biomarkers within the first year after inclusion for prognostic purposes, but could also be used for autoantibody screening as subjected in our present analysis.

### Patients

Patients from ICON-JIA were included in this study, in whom a serum sample beyond the first year after inclusion was available. The samples were analysed at different timepoints after inclusion with a median of 4 years. The timepoints were equally distributed between 0.75 and 7 years after enrollment in ICON.

### Autoantibody screening

The measurements were carried out in the central laboratory of the University Hospital in Muenster. This is a continuously DAkkS-accredited laboratory (DAkkS: German Accreditation Body) with certified assays. Thyroid specific antibodies (Thyroglobulin antibodies: anti-TG, thyroid peroxidase antibodies: anti-TPO, thyroid stimulating hormone receptor antibodies: anti-TSH), celiac specific antibodies (anti-tissue-transglutaminase antibodies: anti-tTG IgA/IgG) and antibodies for connective tissue diseases (CTD screen) were analysed once for each patient. For the determination of anti-TG, anti-TPO and anti-TSH receptor antibody concentrations, the electrochemiluminescence immunoassay (ECLIA) was used on a Cobas e801 instrument (Roche). Total IgA and IgG concentrations were measured using Roche immunoturbidimetry on a Cobas c702 instrument. A fluorescence enzyme immunoassay (FEIA) was used on the Phadia ImmunoCAP 250 platform (Thermo Scientific) for the determination of anti-tTG IgA or IgG as well as the screen for ANA for CTD (connective tissue disease). The ANA-IFT (immunofluorescence assay for ANA) was analyzed at inclusion at the point of care. An additional ANA-IFT was performed on all serum samples with positive CTD screen on HEp2 cells (human epithelial cells supplied by Euroimmun, Luebeck, Germany) with serum dilutions starting at 1:80 and the immunofluorescence results were coded according to ICAP (International Consensus on Antinuclear Antibody Pattern). Specific antibodies were tested according to ICAP codes with fluorescence immunoassays (FIA) covering antibodies against dsDNA, SS-A/Ro, SS-B/La, U1RNP, Sm, CENP-B, Jo-1, Scl-70, Fibrillarin, RNA Polymerase III, ribosomal P-Protein, PM-Scl, PCNA, Mi-2 [14].

### Comparison of autoantibody frequencies

Autoantibody values were examined for differences in gender, age, JIA categories as well as therapy. Other influencing variables, such as elevated immunoglobulins, the presence of ANA-positivity and HLA B27 as reported by the including physicians, or the activity status (active/inactive disease) and the presence of comorbidities were also considered. Finally, in order to investigate whether patients with JIA have higher autoantibody frequencies than the normal population, the observed values in ICON-JIA were compared with those of published historical data from the general population [14–19]. No results were available for the ICON-JIA project’s own control group (consisting of almost 490 healthy participants), as no blood samples were taken from the healthy volunteers for study purposes.

### Statistics

For the statistical analyses, the results were transferred to GraphPad Prism 8. The Fisher exact test was used to check the significance of the results. The association of elevated autoantibody levels with continuous age and JIA disease duration at timepoint of autoantibody screening was analysed by a general linear model with robust error variance. In addition, age and JIA disease duration were dichotomized by median split and group comparisons were carried out by Mann Whitney U-test. Values with *p* < 0.05 were assumed to be significant.

## Results

### Patient characteristics

Of the 499 JIA patients, 333 (66.7%) were female and 166 (33.3%) were male. Furthermore, the patients were divided into two age groups: 2–10 years with 228 patients (45.7%) and 11–22 years with 270 patients (54.3%). The median disease duration was 4.4 years (IQR 3.0 to 6.0) with a median age at onset of 6.0 years (Table 1**)**. Countries of origin for both parents were: 82.8% Europe (*n* = 413), 6.2% Asia (*n* = 31 in total; *n* = 28 Turkish), 0.1% Africa Arab (*n =* 2), 6.0% mixed countries of origin (*n* = 30) and 4.6% unknown (*n* = 23). We could not find a statistically significant association between presence of autoantibodies and disease duration when comparing patients with disease duration of < 4.5 years (*n* = 256) and > 4.5 years (*n* = 240). Regarding the JIA categories, 3% had systemic arthritis, 43% had oligoarthritis (10% extended, 34% persistent) and 5% had psoriatic arthritis. Enthesitis-related arthritis was represented by 10%, seropositive polyarthritis by 2% and seronegative polyarthritis by 28%. Undifferentiated arthritis was present with about 7%. The patient characteristics are shown in Table 1.

**Table 1:** Demographic and clinical characteristics of patients

Patients (n)		499		
**Gender**	Female n (%)	333 (66.7)		
		**median (range)**	**n (%)**	
**Age** (years)	at blood collection	11 [3–22]	**3–10 years**	**11–22 years**
			228 (45.7)	270 (54.3)
	at study inclusion	7 (< 1–17)		
	at onset of disease	6.0 (< 1–16.4)		
		**median (range)**	**n (%)**	
**Disease duration at time of blood collection** (years)		4.4 (0.9–14.1)	**< 4.5 years**	**> 4.5 years**
			256 (51.3)	240 (48.1)
**Disease activity score (cJADAS-10)**		3.0 (0.5–25)		
	no data	*n* = 164		
		**n (%)**		
**Treatment**		**ever before blood collection**	**at time of blood collection**	
	csDMARDs	427 (85.6)	274 (54.9)	
	bDMARDs	206 (41.3)	140 (28.1)	
	Glucocorticoide therapy	442 (84.6)	82 (16.4)	
		**n (%)**		
**JIA categories**	systemic arthritis	16 (3.2)		
	oligoarthritis, extended	49 (9.8)		
	oligoarthritis, persistent	168 (33.7)		
	psoriatic arthritis	27 (5.4)		
	enthesitis-related arthritis	52 (10.4)		
	polyarthritis, seropositive	10 (2.0)		
	polyarthritis, seronegative	142 (28.5)		
	undifferentiated arthritis	34 (6.8)		
	no data	1 (0.2)		
**Physician reported ANA positive patients at inclusion**		310 (62.1)		

csDMARD: conventional synthetic disease-modifying antirheumatic drug; bDMARD: biologic disease-modifying antirheumatic drug

### Autoantibody findings in JIA patients

In total, the investigated autoantibodies were elevated 75 times (15%) in 63 patients (13%). Of these, 40 patients were female, 23 male. In 10 patients (2%) at least 2 and in 2 patients (0.4%) at least 3 autoantibodies were elevated. Overall, anti-TG was elevated in 3% of the patients, with slightly more female than male patients affected. Anti-TPO was elevated in 4% of patients, whereas anti-TSH was not present in any patient. Anti-tTG IgA and anti-tTG IgG were elevated in 2 (female) patients each, of which one patient had both values elevated. One patient had only anti-tTG IgG elevated in presence of normal overall IgA levels. A positive CTD screen was found most frequently with > 7%, in males more frequently than in females. Anti-dsDNA was exclusively found in female patients with a frequency of 2.1% (7 patients). However, there were no statistically significant gender differences overall. With regard to age, the group of 2–10 year-old children was affected slightly more often overall (Table 2). Above all, the CTD screen, at 10.5% (*p* < 0.05), was significantly elevated more than twice as often as in the > 10-year-olds. Younger patients were significantly more frequently positive in the CTD screen. However anti-TPO was significantly more frequent in the older group (p < 0.05). These associations could be confirmed when considering age as continuous parameter in the analysis (CTD screen: relative risk for increase of age by 1 unit 0.95, 95%CI 0.90–0.99, *p* = 0.038; anti-TPO: relative risk for increase of age by 1 unit 1.10, 95%CI 1.01–1.20, *p* = 0.026; anti-TG: relative risk for increase of age by 1 unit 1.16, 95%CI 1.03–1.30, *p* = 0.016).

**Table 2:** Increased autoantibodies depending on gender and age at autoantibody testing

autoantibodies	total (***n*** = 499)		male (***n*** = 166)		female (***n*** = 333)		***p***-value	3–10 years (***n*** = 228)		11–22 years (***n*** = 270)		p-value
anti-TG	15	3.0%	4	2.4%	11	3.3%	0.78	4	1.7%	11	4.3%	0.12
anti-TPO	20	4.0%	7	4.2%	13	3.9%	1	5	2.1%	15	5.9%	**0.04**
anti-tTG IgA	2	0.4%	0	–	2	0.6%	1	1	0.4%	1	0.4%	1
anti-tTG IgG	2	0.4%	0	–	2	0.6%	1	2	0.8%	0	–	0.23
CTD-Screen	36	7.2%	15	9.0%	21	6.3%	0.28	25	10.5%	11	4.3%	**0.01**
anti-dsDNA	7	1.4%	0	–	7	2.1%	0.1	5	2.1%	2	0.8%	0.27
anti-SS-A/Ro	1	0.2%	1	0.6%	0	–	0.33	0	–	1	0.4%	1
anti-SS-B/La	1	0.2%	1	0.6%	0	–	0.33	0	–	1	0.4%	1
anti-Sm	0	–	0	–	0	–	1	0	–	0	–	1
anti-U1RNP	0	–	0	–	0	–	1	0	–	0	–	1
anti-CENP-B	2	0.4%	2	0.1%	0	–	0.11	2	0.8%	0	–	0.23
anti-Jo1	0	–	0	–	0	–	1	0	–	0	–	1
anti-Scl70	0	–	0	–	0	–	1	0	–	0	–	1
≥ 1 autoantibody	63	12.6%	23	13.9%	40	12.0%	0.67	33	13.8%	30	11.8%	0.5
≥ 2 autoantibodies	10	2%	3	1.8%	7	2.1%	1	3	1.3%	7	2.7%	0.34
≥ 3 autoantibodies	2	0.4%	0	–	2	0.6%	1	1	0.4%	2	0.8%	1

### Correlation of autoantibody findings to JIA categories and treatment

**Fig. 1:**
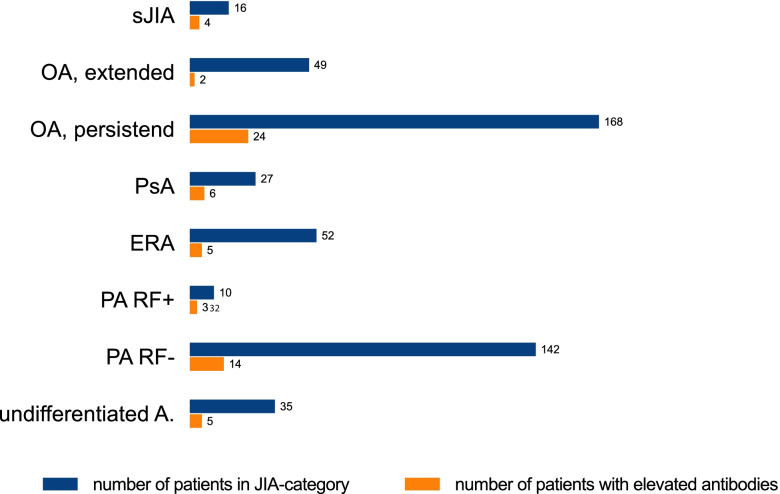
Positive autoantibodies depending on therapy. The blue bars show the overall numbers of patients in the JIA categories according to ILAR classification (total n = 499). The orange bars show the numbers of patients with positive autoantibodies (*n* = 63)

**Fig. 2:**
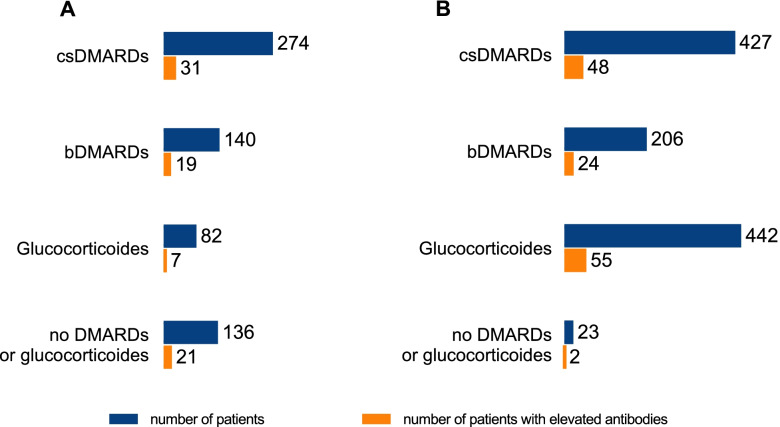
Positive autoantibodies depending on therapy. (A) Treatment at the time of blood collection (blue bars) and the number of patients with positive autoantibodies within the respective groups (orange bars). The patients received either DMARDs, conventional synthetic (csDMARDs) or biological (bDMARDs), glucocorticoids or a combination of drugs from these groups. The patients who were without these medications either received no drug therapy or only nonsteroidal anti-inflammatory drugs (NSAIDs). (B) Treatment at any timepoint ever used before time of blood collection (blues bars) and the number of patients with positive autoantibodies within the respective groups (blue bars)

Autoantibodies were most common in persistent OA, followed by seronegative polyarthritis. However, these categories also included most JIA patients. The proportion of positive tests was highest in systemic arthritis (4 out of 16) and in seropositive arthritis (3 out of 10). However, only a few patients were represented in these two groups, which makes a direct comparison of the JIA categories difficult (Fig. 1). Overall, almost all patients received DMARDs at least once in the course of their treatment. At the time of blood collection 274 patients were treated with conventional synthetic DMARDs (csDMARDs), 140 patients with biological DMARDs (bDMARDs) and 82 with glucocorticoids. An association of autoantibody frequencies with therapy at the time of blood sampling could not be detected (Fig. 2).

### Correlation of autoantibody findings to other patient characteristics

Of the 63 patients (12.6%) with at least one autoantibody detected, over 70% were ANA positive (IFT titer > 1:80) as reported by the physicians at inclusion. Active disease was present in less than half and HLA-B27 was detected in 11.1%. The values did not differ significantly from the patients without autoantibodies.

### Comparison with published data from the general population

The comparison with published literature data showed some differences (Table 3). The prevalence of an elevated anti-TG value in a German population of children was 6.6% and accordingly even higher than in the JIA patients of this study [18]. Anti-TPO frequencies were higher in JIA patients than all other general population control groups. Anti-TSHR was not elevated in any of the JIA patients, but no elevated value was found in population controls either [20]. Anti-tTG IgA was even slightly lower in the ICON-JIA study (0.4%) compared to summarized data from the general population (0.6–1.1%).

**Table 3:** Comparison of ICON-JIA results and data in the general population

	JIA patients	General population					
study	ICON-JIA	Laass et al. [16]	Taubner et al. [18]	Wolf et al. [19]	Mustalahti et al. [17]	Kabelitz et al. [15]	García-García et al. [14]
**year**		2015	2013	2017	2010	2003	2012
**country**	Germany	Germany	Germany	Germany, Austria, United Kingdom	United Kingdom, Italy	Germany	Spain
**Age**	2–22 years (mean 6.8 years)	1–17 years	1–20 years	5 month-18 years (mean 10.2 years)	0–19 years	1–19 years (median 11 years)	1–16 years (mean 8.4 years)
**n (male/female)**	499 (166/333)	12,741 (6546/6195)	670 (351/319)	345 (151/194)	4620 (2271/2349)	660 (293/367)	1387 (710/677)
**anti-TG**	15 (3.01%)		44 (6.6%)				42 (3.0%)
**anti-TPO**	20 (4.01%)	149 (1,2%)	16 (2.4%)			22 (3.4%)	29 (2.1%)
**anti-tTG IgA**	2 (0.4%)	92 (0.7%)		2 (0.6%)	51 (1.1%)		
**anti-tTG IgG**	2 (0.4%)	7 (0.05%)					

### Comparison of clinical and serological comorbidities

In our cohort, a total of 21 (4.2%) clinical comorbidities with autoimmunity (thyroid disease, celiac disease, connective tissue disease) were documented. A total of 8 (1.6%) patients showed serological autoimmunity together with clinically diagnosed autoimmune disease. Clinical comorbidities were already diagnosed in more than half of the patients before the blood test used for our antibody profiling. Autoantibodies without clinical diagnosis were found in 55 (11%) patients (including 2 patients with 2 autoantibody findings). In total, 76 (15.2%) patients were found with clinical or serological autoimmunity (Table 4).

**Table 4:** Overall presence of autoimmune comorbidity or laboratory phenomena

Comorbidity	n	Patients with elevated autoantibodies
**Thyroid specific serology**		
**Hypothyroidism**	**6**	**0**
*diagnosed before analysis*	*2*	
*diagnosed after analysis*	*4*	
**Autoimmune thyroiditis**	**8**	**6**
*diagnosed before analysis*	*5*	*4*
*diagnosed after analysis*	*3*	*2*
**Other thyroid disorder**	**2**	**0**
*diagnosed after analysis*	*2*	
**Without clinical comorbidity**	**21**	**21**
**Celiac disease specific serology**		
**Celiac disease**	**3**	**1**
*diagnosed before analysis*	*3*	*1*
**Without clinical comorbidity**	**2**	**2**
**Connective tissue disease specific serology**		
**Scleroderma**	**1**	**0**
*diagnosed after analysis*	*1*	
**Overlap syndrome**	**1**	**1**
*diagnosed after analysis*	*1*	*1*
**Without clinical comorbidity**	**34**	**34**
**Total patients with clinical comorbidities**	**21**	
**Patients with both clinical comorbidity and antibodies**	**8**	
**Total laboratory results with elevated autoantibodies**	**65**	

## Discussion

An association of JIA with other autoimmune diseases is suspected. However, only few and partially conflicting data on the co-existence of other autoimmune disorders are available. The prevalence of autoantibodies in patients with JIA in Germany is not known. We therefore took advantage of biosamples stored in the ICON-JIA study to analyse laboratory parameters that can indicate autoimmune phenomena in patients with JIA. In the present study, the frequency of thyroid antibodies, celiac serology abnormalities and CTD antibodies in JIA patients in Germany was systematically investigated for the first time.

While there have been a few studies for other countries and ethnic groups investigating the association between autoimmune diseases and JIA, no data were available for the German population. However, since it is known that there are different prevalences for different ethnic groups and also within Europe itself, the results cannot be transferred to the all German JIA patients. In addition, against this background it is not surprising that the individual studies came to very different results. Some of them also included only small samples with a size of 50–150 participants. The large prospective, multicenter cohort studies (ReACCh-Out, CAPS, Nordic Cohort Study), which are comparable to the ICON study, investigated the prognosis, treatment or even influencing factors of JIA very precisely, but not the connection of JIA with other autoimmune diseases. Our study population was comparable to that of other multicenter inception cohorts from Canada, Great Britain and Scandinavia (Denmark, Finland, Sweden, Norway) [21–23]. The sample size, the age at onset and the proportion of female participants are within the range of the other cohorts. The distribution of the JIA categories also largely corresponds to the expected distribution. Minor deviations were noted in this work (ICON-JIA) for systemic arthritis (slightly less frequent at 3%) and seronegative polyarthritis (slightly more frequent at 28%).

Overall, most ICON-JIA values were thus in the range of the general population values. However, a more frequent occurrence in the JIA patients would have been expected, since a connection of JIA was most frequently investigated with celiac disease and in some cases a significantly increased risk was found [5, 6]. Even in the studies that did not find an increased risk of celiac disease, the prevalence was 1.5% [10] and 1.8% [11], which is 3–4 times higher than in our study. This could be in part due to the fact that different celiac antibodies were studied. For example, George et al. studied antigliadin, antireticulin, and antiendomysium antibodies, but not anti-tTG [10]. In addition, most of the samples were very small, ranging from about 50–150 participants. Furthermore, the control groups differed in the different studies. For example, Simon et al. used ADHD patients as a control group, which does not reflect the entire healthy population [7]. Besides, it has to be considered that the patients of this study were at different treatment at time of sampling. This may influence the presence of autoantibodies resulting in a lower level. Anti-tTG IgA and IgG were even higher in controls than in JIA patients. For the CTD screen, no comparable control group was available.

The correlation of JIA and celiac disease is a matter of special interest. However the ESPGHAN guideline (European Society for Paediatric Gastroenterology, Hepatology and Nutrition) defines a risk group (people with certain underlying diseases) with an increased probability of (asymptomatic) celiac disease [24]. JIA is not listed in this category. Since the published studies provided conflicting results, a higher prevalence of celiac disease in JIA patients cannot be clearly assumed. Notably, the samples sizes are partly very small (< 100 study participants) and differ with regard to their ethnicity, and this can influence the prevalence of autoimmune diseases observed even within a region such as Europe [17, 25]. Recently, Lovell et al. compared 2026 patients with JIA and 41572 general pediatric patients via ICD-9 and ICD-10 codes for autoimmune diseases and demonstrated that 14 autoimmune diseases had a significantly higher prevalence in the JIA cohort [24]. In contrast to our study, this study analyzed clinical diagnoses of autoimmune diseases, not autoimmune phenomena. However, the prevalence of clinical autoimmune thyreoiditis among JIA patients was even lower (16/1332; 1.21%) than in our cohort (8/499; 1.60%). Therefore, it appears rather unlikely that this comorbidity was underrecognized in the ICON-JIA patients. For Germany, no results are available for the occurrence of autoantibodies in patients with JIA other than ANA and anti-CCP. It has been discussed whether an association between celiac disease and JIA should prompt us to apply a screening by antibody testing accordingly.

It needs to be noted that the presence of autoantibodies does not prove a clinical diagnosis in patients. However, autoimmune diseases often develop over a longer period of time. It is hypothesized that in genetically predisposed individuals specific autoimmune phenomena can be triggered. In a subclinical phase, autoantibodies can become present. Progressive tissue and organ damage only occurs in the later clinical phase [26]. On the other hand, autoimmune disease may be present even without laboratory proof, and vice versa positive autoantibodies may be present without clinical relevance. In our cohort, a total of 76 patients had either clinically diagnosed autoimmune comorbidity or elevated autoantibodies (15.2%). Of 21 patients with clinical autoimmune comorbidity (especially thyroid autoimmunity), only 8 were also serologically positive at the time of testing, while 55 patients had autoantibodies without clinical diagnosis. In most patients with comorbidity but without autoantibodies, the comorbidity was already noted at inclusion. Autoantibodies may have become negative during therapy. It is conceivable that anti-tissue glutaminase antibodies become negative in celiac disease patients adhering to diet. In our study only one out of three patients with celiac disease was serologically positive, while antibodies were also found in two patients without confirmed celiac disease. On the other hand, a significant number of patients had anti-TPO or anti-TG antibodies despite no thyroid disease recorded. This may warrant a suspicion for the development of autoimmune features in JIA patients as suggested in other reports [3, 27].

There are several limitations to our study. As a major drawback we lack a matched control population. The ICON-JIA control group consisted of non-diseased peers of the JIA patients. For ethical reasons, however, blood sampling from the healthy and especially underage control participants was rejected for study purposes and literature values were used instead. The literature values however, could neither be matched for age nor for ethnic background. It is known that in different ethnic groups partly different prevalence for individual autoimmune diseases may exist [25]. Children and adults are also not equally affected. Autoimmune diseases manifest themselves primarily in adulthood and occur significantly more frequently in this age group (40–50 years of age) [28]. In addition to these demographic differences, each study also has different methodological approaches. For example, the reference values for the individual antibodies are not always identical, which would, however, be more suitable for a direct comparison. Moreover, we only analysed one serum sample per patient, and we might have missed visits at which autoantibodies were present. We choose rather late-stage time-points as we assumed that the development of autoantibodies may evolve over time. In addition, the overall screening of ANA-IFT titers could be considered. We only had ANA-IFT results reported by the physicians at inclusion. Most patients with autoantibodies as tested by us at later time points were ANA-positive at inclusion, but ANA-IFT was not reported for them at the later follow-up time-points. We could only retest those with positive CTD-screen and saw that most of them had elevated ANA, but we do not know the frequency of patients without positive CTD-screen but positive ANA-IFT in the overall cohort. It is conceivable that the CTD-screen is detecting ANA positivity that is often seen in young JIA patients, as opposed to a real predisposition to a connective tissue.

## Conclusion

In conclusion, the frequencies of autoantibodies in JIA patients later than one year in the course of the disease did not differ remarkably from literature data on general populations. A strong general correlation between JIA and laboratory-proven autoimmune phenomena could not be confirmed. It must be noted that there was no matched control group and literature data were used instead. Future studies will be needed to further analyse the relevance of coexisting autoimmune phenomena in JIA patients.

## Supplementary Information

**Additional file 1: Suppl Table S1.** ANA IFT results in patients with positive CTD screen

## Data Availability

The datasets used and/or analysed during the current study are available from the corresponding author on reasonable request.

## Abbreviations

ADHD: Attention deficit hyperactivity disorder
ANA: Antinuclear antibodies
ANA-IFT: Immunofluorescence assay for ANA
anti-TG: Thyroglobulin antibody
anti-TPO: Thyroid peroxidase antibody
anti-TSH: TSH-receptor-antibody
anti-tTG IgA: Anti-tissue transglutaminase antibody against IgA
anti-tTG IgG: Anti-tissue transglutaminase antibody against IgG
bDMARD: Biological DMARD
BMBF: Bundesministerium für Forschung und Bildung (Federal Ministry of Education and Research)
CAPS: Childhood Arthritis Prospective Study
csDMARD: Conventional synthetic DMARD
CTD screen: ANA for connective tissue diseases (CTD)-Screen
DAkks: Deutsche Akkreditierungsstelle (German Accreditation Body)
DMARD: Disease Modifying Antirheumatic Drug
ECLIA: Electrochemiluminescence immunoassay
ERA: Enthesitis related arthritis
ESPGHAN: European Society for Paediatric Gastroenterology, Hepatology and Nutrition
FEIA: Fluorescence enzyme immunoassay
FIA: Fluorescence immunoassays
HEp2 cells: Human epithelial cells
HLA: Human leucocyte antigen
ICAP: International Consensus on Antinuclear Antibody Pattern
ICON-JIA: Inception Cohort of Newly diagnosed patients with juvenile idiopathic arthritis
ILAR: International League of Associations of Rheumatology
JIA: Juvenile idiopathic arthritis
NSAID: Non-steroidal anti-inflammatory drugs
OA: Oligoarthritis
PA RF-: Seronegative polyarthritis
PA RF +: Seropositive polyarthritis
PsA: Psoriatic arthritis
ReACCh-Out: Research in Arthritis in Canadian Children emphasizing Outcome Study
RF: Rheumatoid factor
sJIA: Systemic juvenile idiopathic arthritis
