# Supplementary material for: Prevalence of autoantibodies in patients with juvenile idiopathic arthritis: results from the German inception cohort ICON-JIA
Source: Pediatr Rheumatol Online J. 2022 Feb 2;20:8. doi: 10.1186/s12969-022-00668-9 (PMC8812016; doi:10.1186/s12969-022-00668-9)
Supplement: Supplementary file 1 — Additional file 1: Suppl Table S1. ANA IFT results in patients with positive CTD screen [file 12969_2022_668_MOESM1_ESM.docx]

**Suppl. Table S1:** ANA IFT results in patients with positive CTD screen

| CTD-screen sample | ICAP category | Titer | Antibody (FIA) |
| --- | --- | --- | --- |
| 1 | AC-4 | >1:160 | negative |
| 2 | AC-1+AC-3 | >1:160 | CENPB |
| 3 | AC-1 | >1:160 | negative |
| 4 | AC-4+AC8/9 | >1:160 | negative |
| 5 | AC-1 | >1:160 | negative |
| 6 | AC-1 | >1:160 | dsDNA |
| 7 | AC-5 | >1:160 | negative |
| 8 | AC-2 | >1:160 | negative |
| 9 | Fine+ | 1:80 | negative |
| 10 | AC-1+AC-7 | >1:160 | negative |
| 11 | Fine+ | >1:160 | negative |
| 12 | AC-5 | >1:160 | negative |
| 13 | AC-4 | 1:80 | negative |
| 14 | AC-4 | 1:160 | negative |
| 15 | AC-4 | 1:80 | negative |
| 16 | AC-4 | 1:80 | negative |
| 17 | AC-1 | >1:160 | dsDNA |
| 18 | AC-0 | <1:80 | negative |
| 19 | AC-1 | >1:160 | dsDNA |
| 20 | Fine+ | 1:160 | negative |
| 21 | AC-4 | >1:160 | Ro, La |
| 22 | Fine+ | 1:160 | negative |
| 23 | Fine+ | 1:80 | dsDNA |
| 24 | Fine+ | 1:80 | negative |
| 25 | AC-0 | <1:80 | negative |
| 26 | Fine+AC-5 | 1:160 | negative |
| 27 | AC-5 | >1:160 | negative |
| 28 | AC-4 | 1:80 | negative |
| 29 | Fine+ | 1:160 | negative |
| 30 | Fine+ | 1:160 | dsDNA |
| 31 | Fine+ AC-7 | >1:160 | dsDNA |
| 32 | AC-11+AC-3 | >1:160 | CENPB |
| 33 | AC-1 | >1:160 | dsDNA |
| 34 | AC-1 | >1:160 | negative |
| 35 | AC-4 | >1:160 | negative |
| 36 | AC-4 | >1:160 | negative |
